# Research on energy-saving control of agricultural hybrid tractors integrating working condition prediction

**DOI:** 10.1371/journal.pone.0299658

**Published:** 2024-03-07

**Authors:** Ganghui Feng, Junjiang Zhang, Xianghai Yan, Chunhong Dong, Mengnan Liu, Liyou Xu

**Affiliations:** 1 College of Vehicle and Traffic Engineering, Henan University of Science and Technology, Luoyang, China; 2 State Key Laboratory of Intelligent Agricultural Power Equipment, Luoyang, China; 3 YTO Group Corporation, Luoyang, China; HNBGU: Hemvati Nandan Bahuguna Garhwal University, INDIA

## Abstract

To address the issues of tractors using too much fuel and not being energy efficient, a predictive control strategy based on Pontryagin’s minimum principle integrating working condition prediction is proposed for agricultural hybrid tractors. The Dongfanghong 1804 tractor is being used for research. Firstly, the main parameters of the hybrid drive system are determined and modeled. Secondly, based on the adaptive cubic exponential forecasting method, the working condition information for a period of time in the future is predicted through historical working condition information. Furthermore, combining the predicted working conditions information, the goal is to minimize the total energy consumption cost of the entire machine. Motor power and diesel engine power are control variables. The battery state of charge is a state variable. Subsequently, a predictive control strategy based on Pontryagin’s minimum principle integrating working condition prediction is proposed. Finally, the simulation test is carried out based on the MATLAB simulation platform. Research indicates: under plowing conditions, compared with the power following control strategy, the proposed predictive control strategy can effectively manage the performance of the diesel engine and motor, ensuring they operate at their most efficient level. The total energy consumption costs of the power following control and predictive control strategies are 37.17 China Yuan (CNY) and 33.67 CNY, respectively. The cost of energy used is decreased by 9. 42%, which helps make tractor field plowing more efficient and economical.

## Introduction

Agricultural machinery and equipment are an important foundation for modern agriculture. With the development of new modern agricultural production formats and new production patterns, higher requirements have been put forward in terms of production efficiency, environmental protection, green energy conservation, and other aspects [1–3]. The energy utilization rate of the traditional fuel tractor power system is poor, and the exhaust emissions contain large amounts of pollutants such as CO, carbon oxides, hydrocarbons, and nitrogen oxides, which have become one of the major sources of environmental pollution [4]. Therefore, research on energy-saving and environmentally friendly agricultural vehicles, especially tractors, is of great significance to alleviating the pressure of energy consumption and environmental pollution [5,6]. As a promising energy-saving and environmentally friendly technology, hybrid power system technology is becoming more and more mature in automobile applications [7]. Tractors are the main power machinery of agricultural vehicles. The working conditions are complex, the operating modes need to be changed frequently, and the traction resistance fluctuates frequently. Therefore, the engine load rate is low when the traditional tractor power system is operating, and the engine power cannot be fully utilized [8]. The hybrid system adds a motor, which can optimize the engine’s working area and improve energy utilization efficiency. Therefore, studying the application of hybrid technology in tractors to form a hybrid tractor power system is an important measure to improve tractor fuel economy and reduce exhaust emissions, and is of great significance to energy conservation and environmental protection [9–11].

Energy-saving control strategy is the core technology of hybrid vehicles, which directly determines the vehicle’s fuel economy, power and drivability. It is of great significance to improve the economy and efficiency of vehicles. Optimization of energy-saving control strategies will have a positive impact on the performance and sustainable development for hybrid tractors. At present, energy-saving control strategies are mainly divided into rule-based control strategies and optimization-based control strategies [12–14]. Control strategies that are based on rules have cheap development costs and are simple to put into practice. They are presently used widely in real vehicle control strategies. Xu et al. [15] proposed an energy management strategy based on a control parameter adjustment algorithm to regulate the working status of the diesel engine and the power battery, in response to the relatively high charging status of the power battery in extended-range electric tractors. Simulate and verify based on AVL Cruise simulation platform. The simulation results show that the proposed strategy has good applicability and reduces the equivalent energy consumption of the entire machine. Kim et al. [16] conducted research on predicting the workload of parallel hybrid tractors and proposed an adaptive observer for load torque estimation. The tractor was tested in various working conditions, and the results showed that the fuel economy increased by 3.38%. However, the rule-based control strategy is deterministic and determined based on the designer’s experience and it cannot adapt well to different work conditions. The objective of optimization-oriented control strategies is to lessen or heighten a specified cost function. The cost function is usually a measure of the control objective. Zhu et al. [17] designed a tractor system that uses an engine and a motor as dual power sources and is paired with a hydromechanical continuously variable transmission. For this system, they suggested a method called fuzzy adaptive equivalent fuel consumption minimization strategy. The research found that this control strategy can help divide power more evenly between the engine and the motor, while also keeping the battery state of charge (SOC) balanced. Li et al. [18] designed a real-time adaptive energy-saving control strategy based on stochastic dynamic programming and extreme value search algorithm. The state input control table created offline by stochastic dynamic programming (SDP) is utilized as a control input reference to guarantee inexact worldwide optimality, and an adaptive optimization algorithm-extreme search algorithm is presented to dynamically seek for the nearby most extreme esteem of the framework yield to criticism and redress the SDP input. The driving efficiency of the whole machine has been improved, effectively increasing the tractor operating mileage. However, the fuzzy adaptive equivalent fuel consumption minimization strategy has some shortcomings in practical applications. For example, system identification is inaccurate, parameter adjustment is difficult, real-time computing load is large, and it is sensitive to environmental changes. Real-time adaptive energy-saving control based on stochastic dynamic programming and extreme value search algorithm only guarantees approximate global optimality, and the control strategy is relatively complex.

Through the application of optimized control strategies, the vehicle’s economy has been significantly improved. However, recent reports indicate that overall vehicle efficiency can be further improved by incorporating operating condition predictions. This method was applied earlier to hybrid vehicles. Zhang et al. [19] developed a vehicle velocity forecast model that combines Markov and BP neural networks for plug-in hybrid electric vehicles (PHEV). An adaptive equivalent consumption minimization strategy based on vehicle velocity forecast is posed to optimize driving mode selection and power allocation. The results show that the control strategy enhances the energy consumption economy of PHEV by 3.7% under the same driving conditions. Zeng et al. [20] designed an adaptive equivalent consumption minimization strategy according to demand power prediction optimization for fuel cell hybrid electric vehicles. This strategy regularly renews the optimal equivalence factor through a local optimization process in accordance with the predicted power to focalize the SOC and ensure fuel economy. Simulation results show that the proposed strategy reduces equivalent fuel consumption and is robust to disturbances in power prediction errors.

In view of this, this research focuses on the operating characteristics of agricultural hybrid tractors, combined with the operating differences between tractors and automobiles, to analyze its operating characteristics under rotary tillage conditions. Accordingly, an energy-saving control strategy integrating working condition prediction was proposed to better improve the energy consumption economy of the tractor [21–24]. This research provides a new method to improve the energy utilization efficiency of tractors. The main contributions are listed below: (a) For tractor plowing conditions, a working condition prediction strategy based on the adaptive cubic exponential forecasting method is designed to predict working condition information for a period of time in the future through historical working condition information. (b) A predictive control strategy (PCS) based on Pontryagin’s minimum principle that integrates working condition prediction is proposed to reduce the energy consumption of tractor plowing. The results of the study show that the proposed control strategy has high accuracy in predicting the tractor plowing conditions. The total cost of energy consumption of the hybrid tractor was reduced by 9.42% under plowing conditions.

This article is structured as follows. Firstly, the overall drive system of the agricultural hybrid tractor is explained. Taking the Dongfanghong 1804 tractor as the research object, the main parameters of its parallel hybrid drive system were determined. Then, the components of the hybrid tractor are modeled. On the basis of the tractor simulation model, a PCS based on the Pontryagin’s minimum principle integrating working condition prediction is designed. The power following control strategy (PFCS) is used for comparison. Lastly, through MATLAB simulation, the superiority of PCS was verified and the conclusions of this study were obtained.

## Materials and methods

Firstly, the hybrid tractor drive system structure and its main component parameters in this study were explained. Secondly, the main components of the tractor were modeled. Finally, the control strategy was designed based on the tractor model.

### Tractor drive system and main parameters

According to the structure of the agricultural hybrid tractor drive system and the working requirements of the Dongfanghong 1804 tractor, transmission parameters, and other main tractor performance parameters are determined.

#### Drive system structure

**Fig 1:**
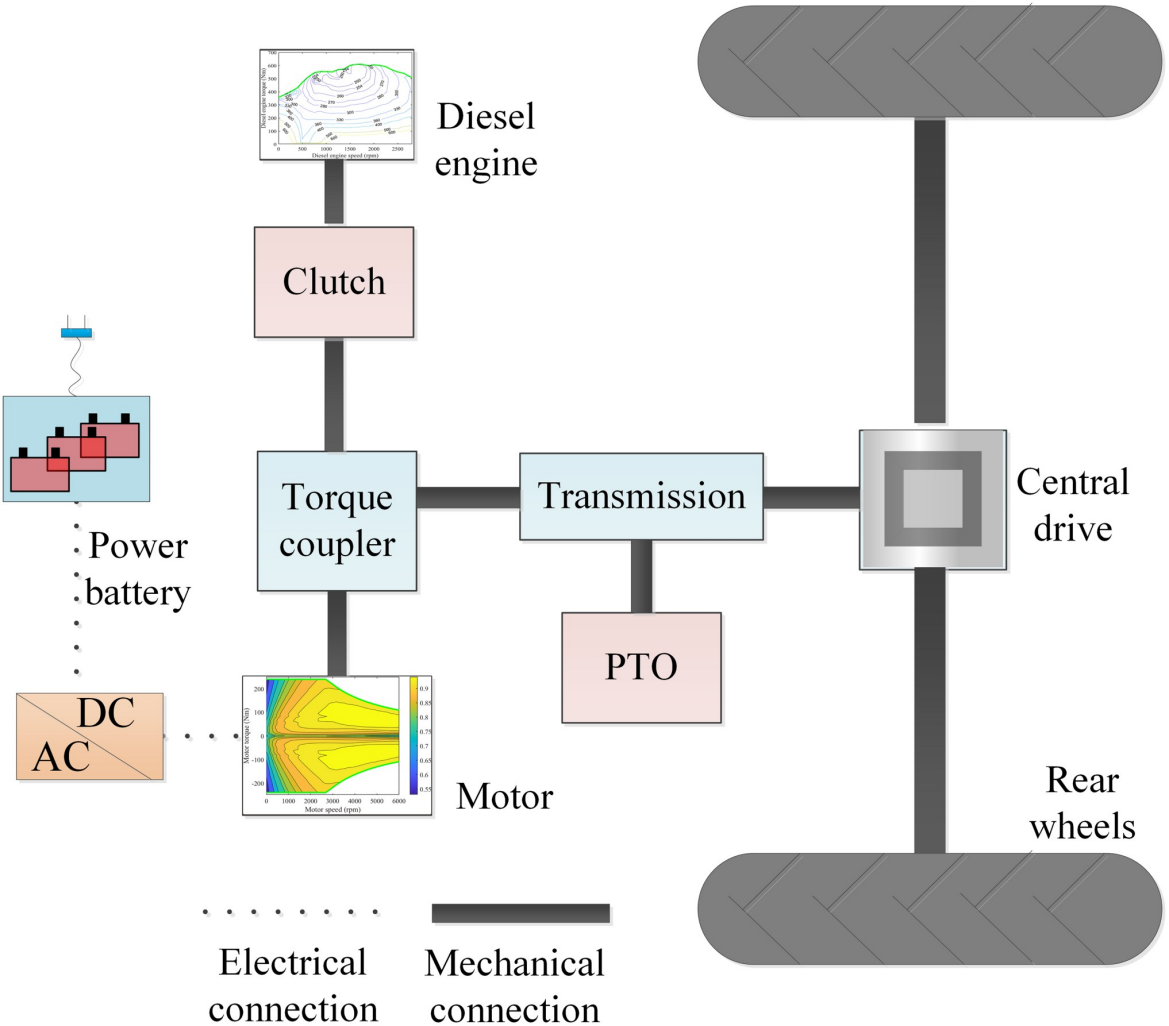
Parallel agricultural hybrid tractor drive system structure diagram.

The structure of the parallel agricultural hybrid tractor drive system is shown in Fig 1. This is a parallel hybrid tractor, powered by a motor and a diesel engine. The major components involve power battery, motor, diesel engine, power take-off (PTO), torque coupler, transmissions, and central drive.

Both the diesel engine and the motor can be driven individually or simultaneously to provide power for the tractor. The motor and diesel engine transmit the output torque to the input shaft of the transmission through the torque coupling, thereby driving the transmission to output power, which is taken as the power input of the central drive and the PTO power output shaft, respectively.

#### Transmission parameters

Tractors can be divided into operating modes and transportation modes according to different uses. As a power machine, they mainly provide traction work. The transmission essentially achieves torque conversion through speed changes, and meets the changing traction resistance by changing the traction force of the tractor under different operating conditions [25]. The transmission gears should be designed in the light of the load under different working conditions to improve operating efficiency and power utilization.

According to the working characteristics of the agricultural tractor and the characteristics of the hybrid tractor driving system, the central drive ratio and the gearbox transmission ratio are designed. And calculate the theoretical velocity of each tractor gear [26,27]. The particular parameters of the transmission are appeared in Table 1.

**Table 1:** Transmission ratios of each gear and their theoretical velocity.

Items	Transport III	Transport II	Transport I	Operation II	Operation I	Amble II	Amble I
Transmission ratio	0.862	1.277	2.327	3.366	4.963	7.425	11.238
Theoretical velocity (km/h)	36.98	26.768	17.364	11.977	7.654	5.231	3.502

#### Main performance parameters of agricultural hybrid tractors

With reference to the Dongfanghong 1804 tractor, combined with the actual production conditions of the parts manufacturer and the hybrid system structure, select the main component parameters of the hybrid tractor [28]. This study takes a 132 kW tractor as the design prototype, using a diesel engine as the main power source and a motor as the auxiliary power source. According to the common characteristic of "peak shaving and valley filling" in hybrid tractors, the diesel engine is mainly suitable for pure diesel engine working mode under medium and low load operations and hybrid working mode under medium and high load operations. The diesel engine provides the stable power required for these tasks, while the motor provides the dynamic demand power. Therefore, the mixing degree is determined to be 20%, which means the power ratio of the diesel engine and motor is 8:2. The particular parameters are shown in Table 2.

**Table 2:** Particular parameters of agricultural hybrid tractor.

Items	Parameters	Value (Unit)
Tractor	Quality of use Drive wheel radius	7200 (kg) 0.785 (m)
Diesel engine	Rated speed Rated power Peak torque	2200 (rpm) 105 (kW) 610 (Nm) (1600 rpm)
Motor	Rated (peak) speed Rated (peak) power Rated (peak) torque	2800(6000) (rpm) 27(70) (kW) 92(240) (Nm)
Power battery	Capacity Rated voltage SOC	45 (Ah) 320 (V) 0.20–0.90
Central drive	Transmission ratio	18.60

### Drive system modeling

According to the drive system of the agricultural hybrid power tractor, the models of its main components are established, including the powertrain model, the dynamics model of the plow unit, and the power component model. In the end, the tractor simulation model is built.

#### Powertrain model

Agricultural hybrid tractors require power from electric motors and diesel engines. Use the input of the torque coupler to calculate the required power for the entire machine. The calculation equation is as follows:

$$\begin{array}{cc} P_{req} & =P_{mreq}+P_{ereq}\\ \; & =P_{m}\eta_{m}+P_{e}\eta_{e} \end{array}$$ (1)

where *P*_*req*_ denotes the torque coupler input power; *P*_*mreq*_ and *P*_*ereq*_ denote the required power of the diesel engine and motor, respectively; *P*_*m*_ and *η*_*m*_ denote the power and efficiency of the motor, respectively; *P*_*e*_ and *η*_*e*_ are the power and efficiency of the diesel engine, respectively.

Calculate the power source speed based on the tractor operating velocity and the parameters of each component. Using the speed of the diesel engine as the speed of the power source, the calculation formula is:

$$n_{e}=nreq=n_{tire}i_{b}i_{zy}$$ (2)

where

$$n_{tire}=\frac{v}{0.377r}$$

where *n*_*tire*_ denotes the driving wheel speed; *n*_*e*_ and *n*_*req*_ represent the diesel engine speed and the torque coupler input demand speed, respectively; *v* is the tractor working velocity; *r* represents the driving wheel radius; *i*_*b*_ and *i*_*zy*_ are the transmission ratios of the transmission and the central drive, respectively.

#### Dynamic model of plowing unit

When the tractor is plowing in the field, the driving force demands to endure the resistance of the tractor pulling agricultural implements and other driving resistance. At low velocity, when the tractor is in working, the impact of acceleration resistance and air resistance on the tractor can be disregarded [29]. The relationship between the driving force and various resistances is as follows:

$$F_{TN}=F_{r}+F_{f}+F_{p}$$ (3)

where *F*_*TN*_ represents the tractor driver; *F*_*r*_, *F*_*f*_, and *F*_*p*_ denote the tillage resistance, rolling resistance, and grade resistance of the tractor, respectively.

When the agricultural hybrid tractor pulls the plowing unit, the required power at the input end of the torque coupler is calculated as follows:

$$P_{req}=\frac{F_{TN}v}{\eta_{zy}\eta_{b}\eta_{o}}$$ (4)

where *η*_*o*_, *η*_*b*_, and *η*_*zy*_ denote the efficiency of torque coupling, transmission, and central transmission, respectively; *v* is the tractor working velocity.

#### Power component model

The power component models of hybrid tractors mainly include tire model, power battery model, motor model and diesel engine model. Tire model is the end of a tractor’s power output. The tractor moves by driving the wheels to rotate. The power battery model is the energy storage device of the motor model, which can provide energy for the motor and also store the energy generated by the motor. Diesel engine model and motor model are the power sources for hybrid tractors. The model building process of dynamic components has been described in previous research [30], and this part is not the focus of this paper. Therefore, only a brief description of the modeling results is given here.

**Fig 2:**
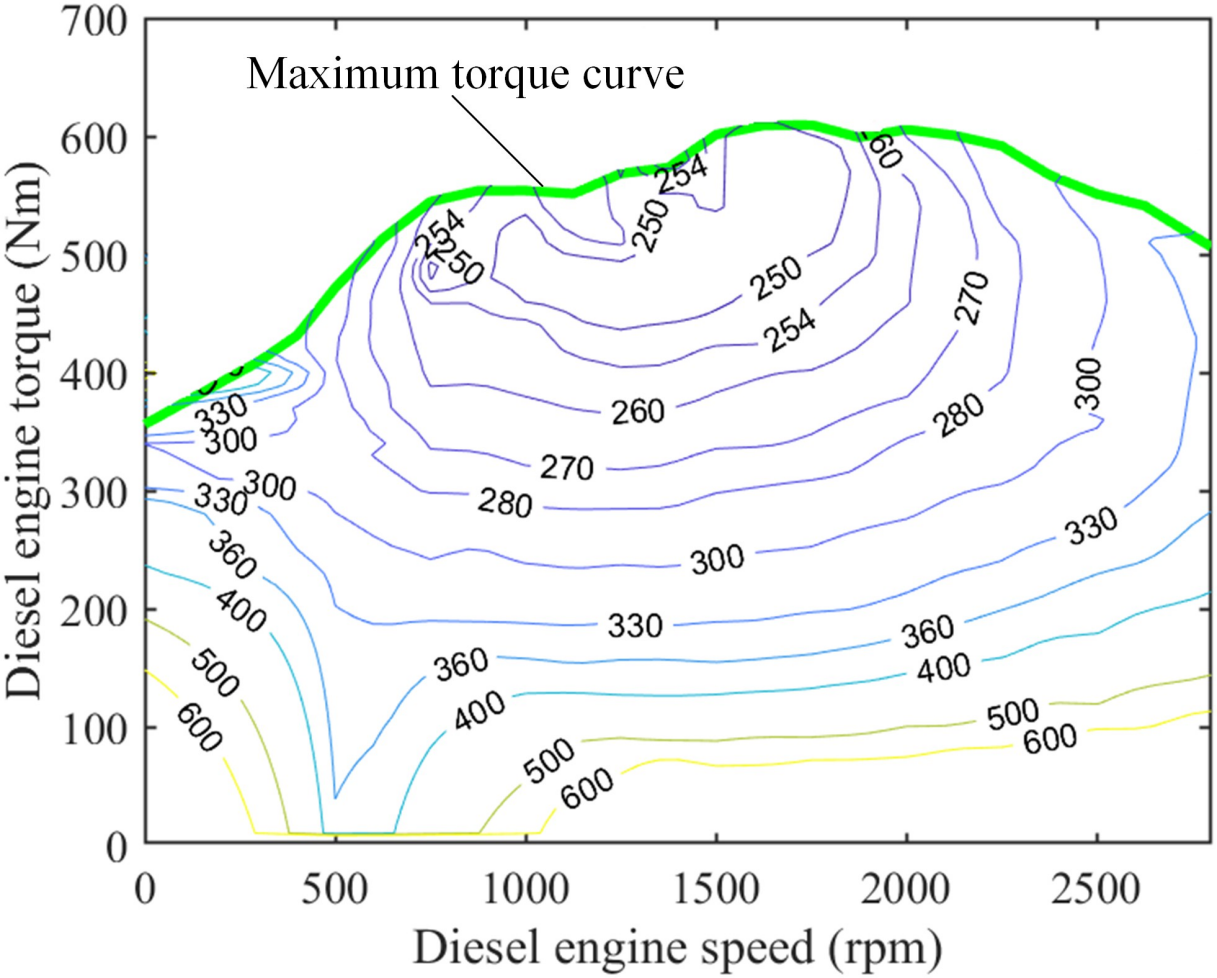
MAP diagram of diesel engine numerical model.

**Fig 3:**
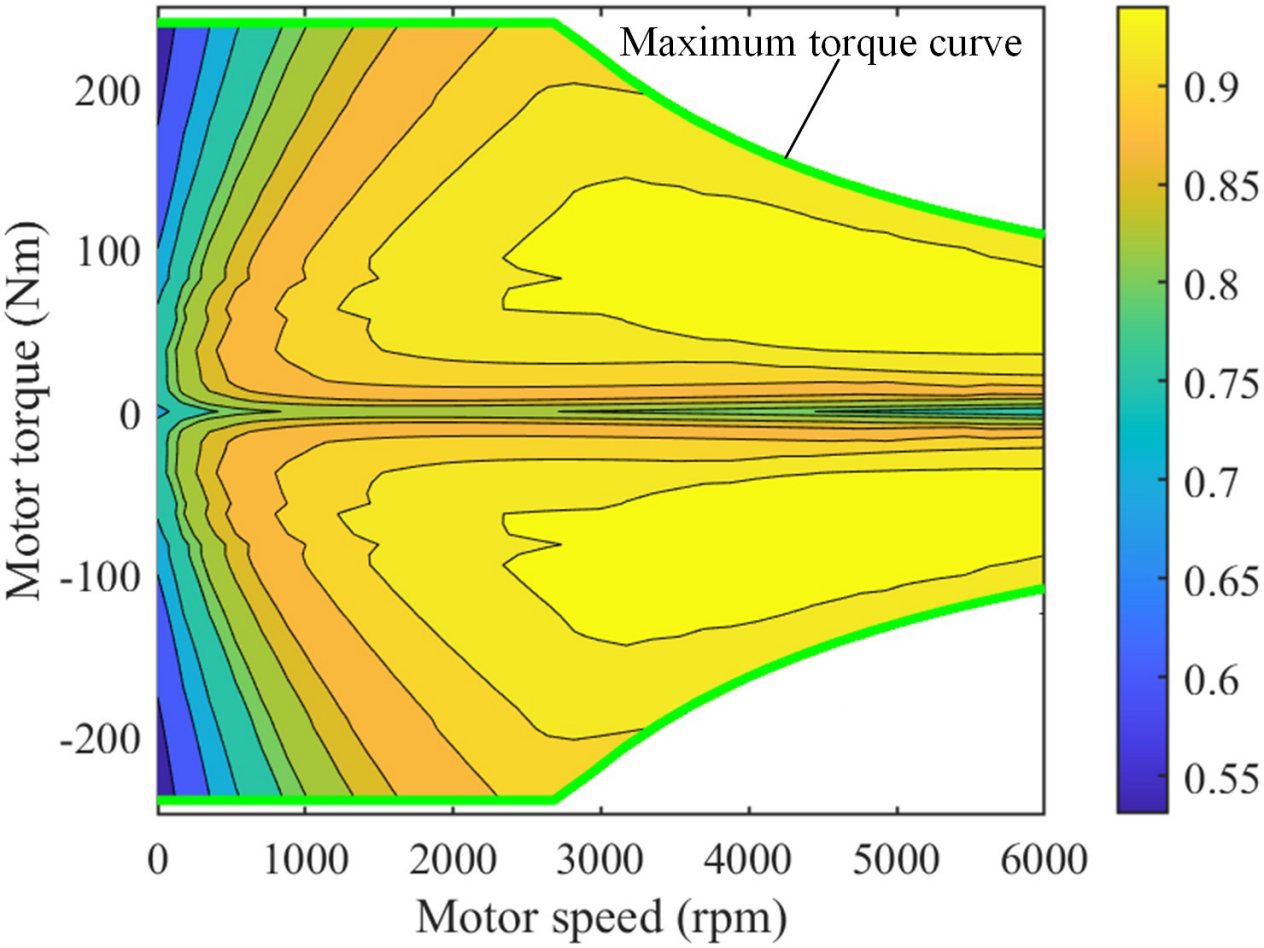
MAP diagram of motor numerical model.

The tire model adopts Duggof model. The diesel engine model and motor model adopt numerical models, as shown in Figs 2 and 3.

It can be seen from Fig 2 that the fuel consumption rate of diesel engine is a functional relationship with torque and speed as independent variables. The relationship is as follows:

$$m_{e}=f_{e}\left(T_{e},n_{e}\right)$$ (5)

where *T*_*e*_ and *m*_*e*_ represent the torque and fuel consumption rate of the diesel engine, respectively.

The calculation formula of motor efficiency can be expressed as follows:

$$\eta_{m}=f_{m}\left(T_{m},n_{m}\right)$$ (6)

where *T*_*m*_ and *n*_*m*_ are the torque and speed of the motor, respectively.

**Fig 4:**
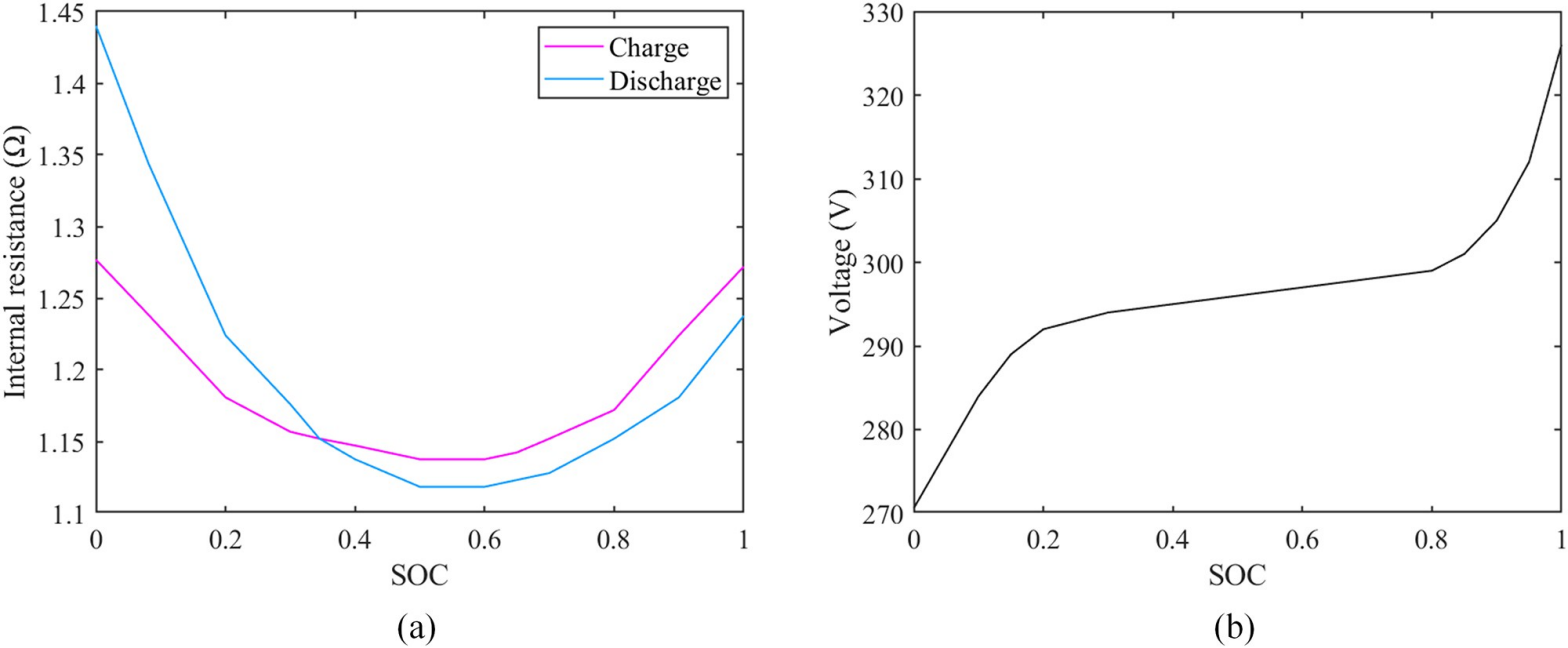
The relationship between power battery characteristics and SOC. (a) The relationship curve between the charging or discharging internal resistance of the power battery and the SOC; (b) The relationship curve between power battery electromotive force and SOC.

The power battery adopts the equivalent circuit R-int model. Ignore the effect of temperature on power batteries. The relationship between the external characteristics of the power battery and SOC is determined through experiments, as shown in Fig 4.

The power required by the power battery is as follows:

$$P_{b\text{at}}=\left\{\begin{array}{l} \frac{P_{m}}{\eta_{bat}},P_{m}\geq 0\\ P_{m}\eta_{bat},P_{m}<0 \end{array}\right.$$ (7)

where *P*_*bat*_ and *η*_*bat*_ represent the power and efficiency of the power battery, respectively. When *P*_*m*_ is greater than 0, it means that the power battery is discharged. On the contrary, it represents the charging of the power battery.

The calculation equation of the entire loop current is shown below:

$$I_{b}=\frac{E_{0}-\sqrt{{E_{0}}^{2}-4R_{0}P_{m}}}{2R_{0}}$$ (8)

where *I*_*b*_ and *E*_0_ represent the output current and electromotive force of the power battery, respectively; *R*_0_ denotes the internal resistance of the power battery.

The ampere-hour integration modus is utilized to calculate the alteration in the SOC value of the power battery. The calculation formula is:

$$SOC(t)=SOC_{0}-\frac{\int_{0}^{t}I_{b}(t)dt}{Q_{b}}$$ (9)

where *SOC*_0_ denotes the initial value of SOC; *Q*_*b*_ denotes the rated capacity of the power battery.

#### Tractor simulation model

**Fig 5:**
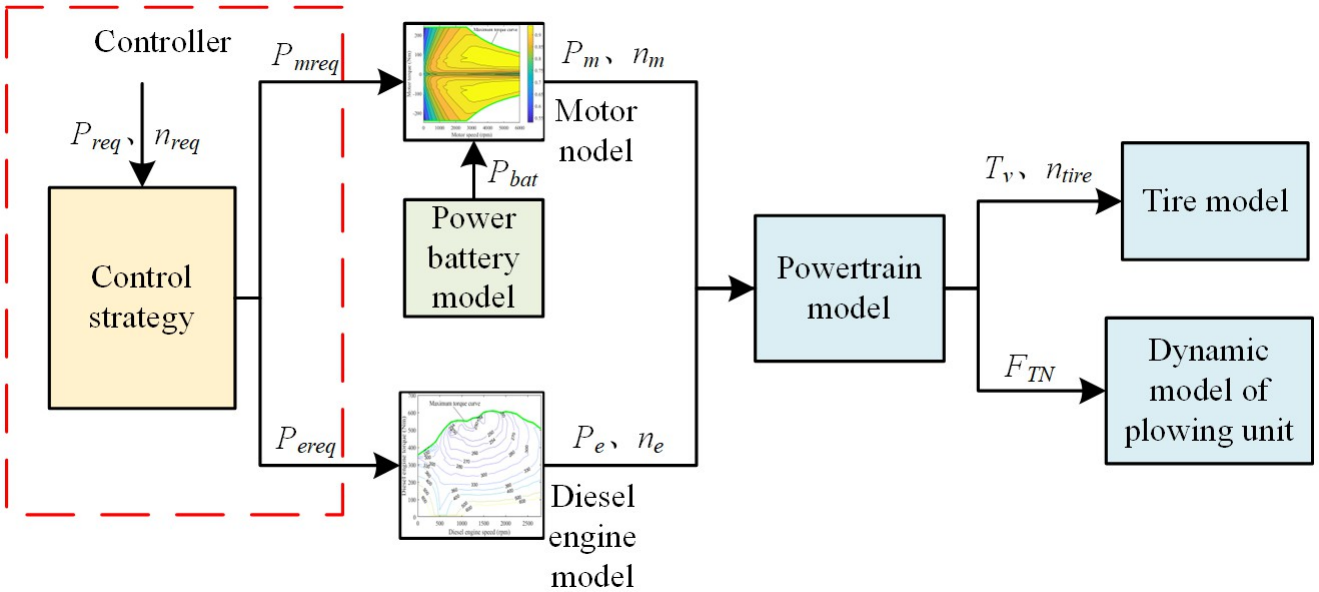
Schematic diagram of tractor simulation model.

Combined with the characteristics of the agricultural hybrid tractor drive system, a tractor simulation model was built based on MATLAB, as shown in Fig 5.

The simulation model includes the plowing unit dynamics model, diesel engine model, motor model, powertrain model, tire model and battery model. According to the working conditions of the tractor, the controller collects signals and calculates and processes them according to Eqs (1), (2) and (4) to obtain the required power and required speed (*P*_*req*_ and *n*_*req*_) of the tractor. Then, the controller allocates the corresponding required power (*P*_*mreq*_ and *P*_*ereq*_) to the motor and diesel engine according to the established control strategy. The motor and diesel engine run according to the instructions, output the definite power and speed (*P*_*m*_, *n*_*m*_, *P*_*e*_, and *n*_*e*_) and deliver the power to the tire model (*T*_*v*_ and *n*_*tire*_) and the plowing unit dynamics model (*F*_*TN*_). Simultaneously, the battery model supplies energy (*P*_*bat*_) based on the power demand of the motor model.

### Control strategy design

In order to predict tractor plowing conditions, a working condition prediction strategy based on the adaptive cubic exponential forecasting method is designed. Then, combined with the working condition prediction information, an energy-saving control strategy based on Pontryagin’s minimum principle that integrates working condition prediction is proposed. Reduce energy consumption of tractor plowing operations.

#### Adaptive cubic exponential forecasting method

According to the number of smoothing, the exponential smoothing method can generally be divided into one-time exponential smoothing method, two-time exponential smoothing method and three-time exponential smoothing method. However, the first-order exponential smoothing method is appropriate for forecasting and analyzing time series without any trend effect and with a gradual trend, whereas the second-order exponential smoothing method is primarily suitable for predicting time series with linear changes. Due to the irregularity of tractor velocity and plowing resistance changes, the trend is nonlinear. Therefore, it is more suitable to use cubic exponential smoothing method for prediction.

**Fig 6:**
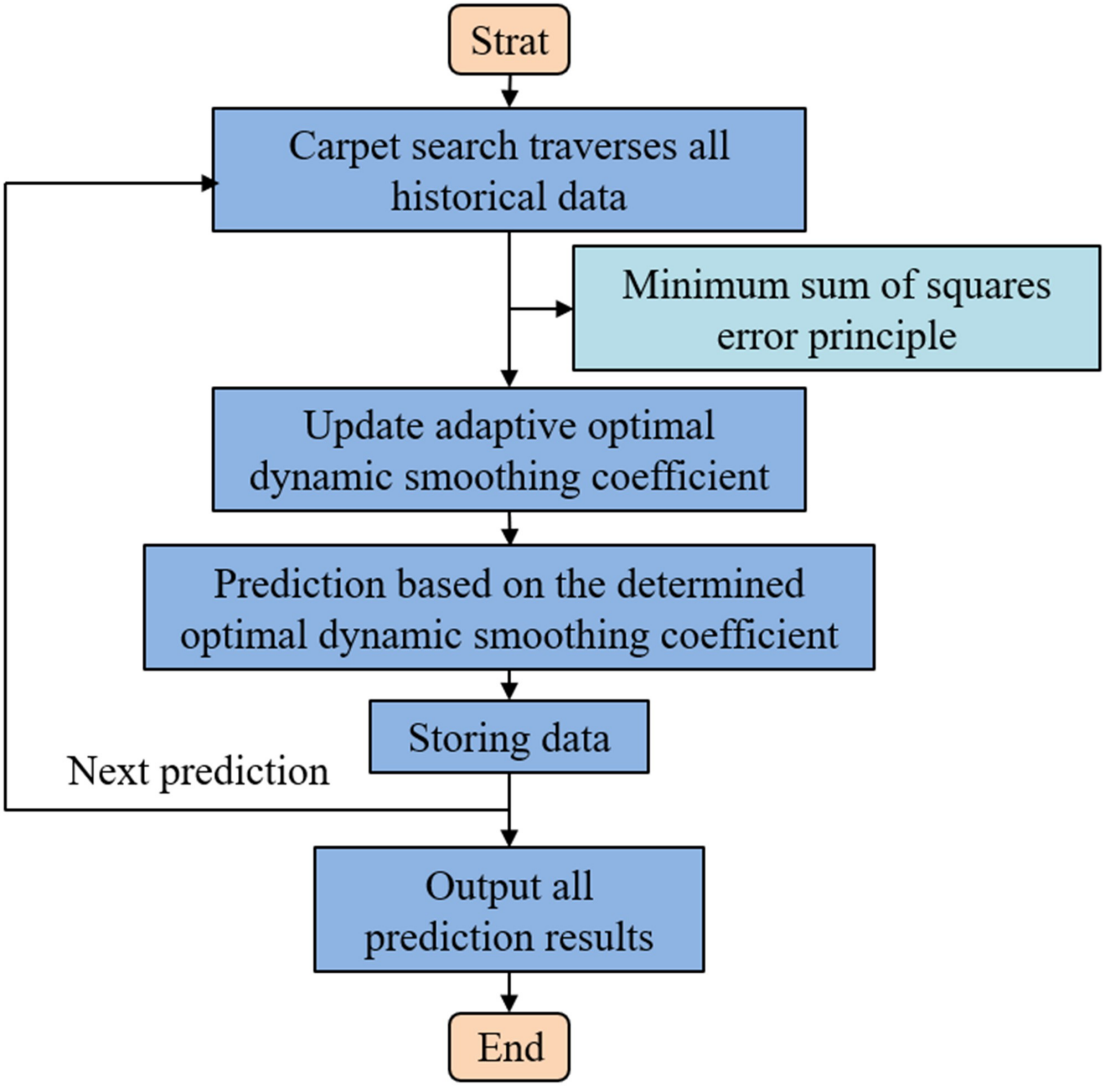
Adaptive cubic exponential forecasting method.

When using the exponential smoothing method for actual prediction, the smoothing coefficient is static. If the time series data changes significantly, the adaptability of the prediction model will decrease, thus affecting the prediction accuracy. For this purpose, a modified dynamic cubic exponential smoothing method is used. The smoothing coefficient can continuously follow changes in time series data and automatically make corresponding adjustments [31]. This is the adaptive cubic exponential forecast method, and the specific process is shown in Fig 6.

1. The adaptive exponential forecast method alludes to the utilize of carpet traversal search algorithm to optimize the smoothing coefficient. Get the dynamic smoothing coefficient for prediction. The dynamic smoothing coefficient is calculated as follows:
$$\varepsilon_{k,t}=\frac{\beta_{k}}{1-{\left(1-\beta_{k}\right)}^{t}}$$ (10)
  where *ε*_*k*,*t*_ is the dynamic smoothing coefficient in the *k*-th prediction, (0<*ε*_*k*_ <1); *β*_*k*_ denotes the still smoothing coefficient in the *k*-th prediction.
2. Smoothing of historical data.
$$\left\{\begin{array}{l} S_{k,t}^{\text{(1)}}=\varepsilon_{k,t}X_{k,t}+(1-\varepsilon_{k,t})S_{k,t-1}^{\text{(1)}}\\ S_{k,t}^{\text{(2)}}=\varepsilon_{k,t}S_{t}^{\text{(1)}}+(1-\varepsilon_{k,t})S_{k,t-1}^{\text{(2)}}\\ S_{k,t}^{\text{(3)}}=\varepsilon_{k,t}S_{t}^{\text{(2)}}+(1-\varepsilon_{k,t})S_{k,t-1}^{\text{(3)}} \end{array}\right.$$ (11)
  where $S_{k,t}^{\left(1\right)}$,$S_{k,t}^{\left(2\right)}$, and $S_{k,t}^{\left(3\right)}$ represent the first, second, and third exponential smoothing values of the *k*-th prediction of the *t* time data, respectively; *X*_*k*,*t*_ denotes the *k*-th prediction of actual data at time *t*.
3. Evaluation of prediction accuracy. Common prediction accuracy evaluation indicators include the average absolute value of errors, sum of squares of errors, and mean square error. This study adopts the principle of minimum sum of squares of errors, which is easier to calculate.
$$f_{k}=\text{min}\sum_{i=k}^{k+N-1}(Y_{k,t}-X_{k,t}{)}^{2}$$ (12)
  where *f*_*k*_ denotes the sum of squared errors; *Y*_*k*,*t*_ and *X*_*k*,*t*_ represent the prediction value and actual value of the *k*-th prediction data, respectively; *N* denotes the total number of periods.
4. Predictions of future data. *a*_*k*,*t*_, *b*_*k*,*t*_, and *c*_*k*,*t*_ denote the prediction coefficients of the *k*-th, calculated as follows:
$$\left\{\begin{array}{l} a_{k,t}=3S_{k,t}^{\left(1\right)}-3S_{k,t}^{(2}+S_{k,t}^{\left(3\right)}\\ b_{k,t}=\frac{\varepsilon_{k,t}}{2{\left(1-\varepsilon_{k,t}\right)}^{2}}((6-5\varepsilon_{k,t})S_{k,t}^{\left(1\right)}-(10-8\varepsilon_{k,t})S_{k,t}^{\left(2\right)}+(4-3\varepsilon_{k,t})S_{k,t}^{\left(3\right)}\\ c_{k,t}=\frac{\varepsilon_{k,t}^{2}}{2{\left(1-\varepsilon_{k,t}\right)}^{2}}(S_{k,t}^{\left(1\right)}-2S_{k,t}^{\left(2\right)}+S_{k,t}^{\left(3\right)}) \end{array}\right.$$ (13)

The predicted value of the *k*-th in period *t* + *T*_*ace*_ is:

$$Y_{k,t+T_{ace}}=a_{k,t}+b_{k,t}T_{ace}+c_{k,t}T_{ace}^{2}$$ (14)

Different from the static smoothing coefficient *β*, the dynamic smoothing coefficient *ε*_*k*,*t*_ is a function of the period *t*. As the number of predictions *k* and the number of prediction periods *t* change, it can track the trend of time series fluctuations over time. Improved adaptability and thus improved prediction accuracy.

#### Energy-saving control strategy integrating working condition prediction

Agricultural hybrid tractors have two energy sources: electric energy and fuel. In order to unite energy, the economic function of energy consumption is defined as the sum of the cost of electrical energy and the cost of diesel fuel. The total energy consumption cost during tractor operation is as follows:

$$Q_{c}(t)=\int_{0}^{t_{f}}\left(j_{e}Q_{f}(t)+\frac{j_{m}P_{bat}(t)}{\eta_{bat}}\right)dt$$ (15)

where

$$Q_{f}(t)=\frac{f_{e}P_{e}}{1000\times 3600\times 0.84}$$

where *Q*_*c*_(*t*) denotes the total energy cost of tractor operation; *j*_*m*_ and *j*_*e*_ denote the prices per kilowatt hour of electricity and per liter of oil, respectively; *Q*_*f*_(*t*) represents the fuel consumption at time *t*; *f*_*e*_ is the diesel engine fuel consumption rate at time *t*.

Since the operational capabilities of components are restricted by practical situation, the following constraints need to be met.

$$\left\{\begin{array}{l} P_{m\text{min}}\leq P_{m}(t)\leq P_{m\text{max}}\\ P_{e\text{min}}\leq P_{e}(t)\leq P_{e\text{max}}\\ SOC_{\text{min}}\leq SOC(t)\leq SOC_{\text{max}} \end{array}\right.$$ (16)

where *P*_*e*min_, *P*_*e*max_, *P*_*m*min_, and *P*_*m*max_ are the maximum power and minimum power allowed by the diesel engine and motor at time *t*, respectively; *SOC*_max_ and *SOC*_min_ denote the maximum and minimum values allowed by SOC, respectively.

Eq (16) constitutes the allowable reachable set *R* of the control variables.

The Pontryagin’s minimum principle is used to control the work of the motor and diesel engine. Taking the SOC value as the state variable, the diesel engine power *P*_*e*_ and motor power *P*_*m*_ as the control variables, introducing the co-state variable *ζ*, the Hamilton function is constructed as follows:

$$\begin{array}{cc} H(x,u,\zeta ,t) & =Q_{f}(t)-\zeta (t)\frac{j_{m}S\dot{O}C(t)}{j_{e}\eta_{bat}}\\ \; & =Q_{f}(t)-\zeta (t)\frac{j_{m}}{j_{e}\eta_{bat}}\frac{U_{b}(t)-\sqrt{U_{b}^{2}-4P_{b}(t)R_{0}(t)}}{2R_{0}(t)Q_{b}} \end{array}$$ (17)

where *x* and *u* represent the state variables and control variables, respectively; *ζ* denotes the pending Lagrange multiplier.

The regular equation is as follows:

$$\begin{array}{cc} \dot{\zeta }(t) & =-\frac{\partial H(SOC,u,\zeta )}{\partial SOC}\\ \; & =\zeta (t)\frac{j_{m}}{j_{e}\eta_{bat}}\frac{\partial SOC(t)}{\partial SOC} \end{array}$$ (18)

Under normal circumstances, power battery voltage and resistance characteristics are related to the battery itself, and their dependence on SOC in a short period of time is negligible. This article assumes that the power battery current change rate is approximately 0, *ξ*(*t*) = 0. Then, it can be obtained by solving the regular equation:

$$\begin{array}{cc} \zeta (t) & =\zeta \left(t_{0}\right)\\ \; & =\zeta_{0} \end{array}$$ (19)

The state equation is:

$$x(t)=\overset{\cdot }{SOC}(t)$$ (20)

where

$$\begin{array}{cc} S\dot{O}C(t) & =-\frac{I_{b}(t)}{Q_{b}}\\ \; & =-\frac{U_{b}(t)-\sqrt{U_{b}^{2}-4P_{b}(t)R_{0}(t)}}{2R_{0}(t)Q_{b}} \end{array}$$

The boundary conditions are as follows:

$$\left\{\begin{array}{l} SOC\left(t_{0}\right)=SOC_{0}\\ SOC\left(t_{f}\right)=SOC_{f} \end{array}\right.$$ (21)

The minimum conditions are as follows:

$$H\left[x^{*}(t),\zeta (t),u^{*}(t)\right]=\underset{u\in R}{\text{min}\;H}\left[x^{*}(t),\zeta (t),u(t)\right]$$ (22)

The optimal control variables are:

$$u^{*}=\text{arg}\;\underset{u\in R}{\text{min}}[x(t),\zeta (t),u(t)]$$ (23)

**Fig 7:**
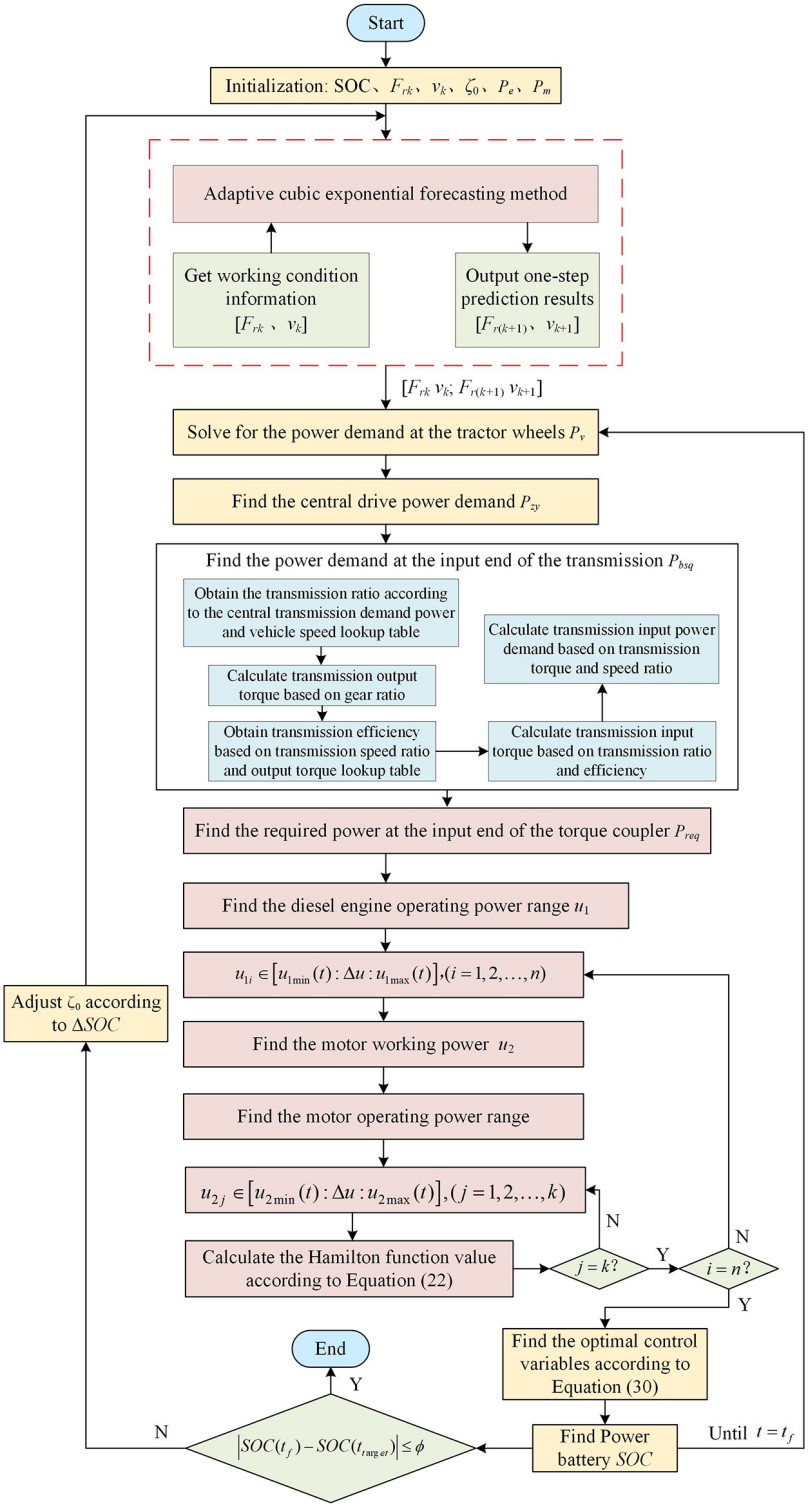
Predictive control strategy solution flow chart.

The PCS is an integration of adaptive cubic exponential forecasting and Pontryagin’s minimum principle. The adaptive exponential prediction method predicts vehicle driving information and provides vehicle driving information for the Pontryagin’s minimum principle. The adaptive exponential forecasting method predicts vehicle driving information and provides vehicle driving information for the Pontryagin’s minimum principle. Pontryagin’s minimum principle uses a cyclic iteration modus to legitimately optimize the operational power of the motor and diesel engine to minimize the total energy consumption cost of the entire machine. The PCS process is illustrated in Fig 7.

1. First, initialize the tractor data and Lagrangian factor *ζ*_0_. Then, Then, collect historical working condition information (farming resistance *F*_*rk*_ and vehicle velocity *v*_*k*_). Predict working condition information for a period of time in the future based on the adaptive cubic exponential forecast method. Subsequently, the predicted data is output. During this process, one-step prediction data is the most precise, therefore, one-step prediction is used. Only one step of prediction is performed here, that is, [*F*_*r*(*k*+1)_ *v*_*k*+1_] is obtained from [*F*_*rk*_ *v*_*k*_] through the prediction method. Finally, the current data and predicted data are passed to the next stage to provide data support for energy-saving control ([*F*_*rk*_ *v*_*k*_; *F*_*r*(*k*+1)_ *v*_*k*+1_]).
2. From the data [*F*_*rk*_ *v*_*k*_; *F*_*r*(*k*+1)_ *v*_*k*+1_], the power demand at the wheels of the complete machine is calculated according to the dynamic equation.
$$P_{v}=F_{TN}v$$ (24)
  where *P*_*v*_ is the power demand at the wheels of the tractor.
3. According to the power demand at the wheels of the entire machine, the power demand of the central transmission is obtained.
$$P_{zy}=\frac{P_{v}}{\eta_{zy}}$$ (25)
  where *P*_*zy*_ represents the power demand of the central drive.
4. Find the required power *P*_*bsq*_ at the transmission input end. First, obtain the transmission ratio based on the central drive power demand and the tractor speedometer. Then, the transmission output torque is calculated based on the known speed ratio. Next, obtain the transmission efficiency through the transmission ratio and torque lookup table. The transmission input torque is then calculated based on the transmission speed ratio and efficiency. Finally, the torque and speed ratio are used to calculate the power required at the transmission input.
5. Find the required power *P*_*req*_ at the input end of the torque coupler. According to the torque and speed at the transmission input end, look up the table to obtain the torque coupling efficiency. Then, based on the speed ratio and efficiency of the transmission, the torque at the input end of the transmission is calculated. Finally, the power required at the input of the torque coupler is calculated based on the torque and speed ratio.
6. According to the power at the input end of the torque coupler, find the value range of the diesel engine operating power.
$$\left\{\begin{array}{l} u_{1\text{max}}(t)=P_{e\text{max}}(t)\\ u_{1\text{min}}(t)=0 \end{array}\right.$$ (26)
  where *u*_1max_ and *u*_1min_ represent the maximum and minimum power allowed by the diesel engine at time *t*, respectively.
7. Within the value range, the operational power of the diesel engine is discretized with a step size Δ*u*.
$$u_{1i}\in \left[u_{1\text{min}}(t):\Delta u:u_{1\text{max}}(t)\right],(i=1,2,\ldots ,n)$$ (27)
8. Find the operational power of the motor and determine its value range.
$$u_{2}(t)=P_{req}(t)-u_{1i}$$ (28)
  where
$$\left\{\begin{array}{l} u_{2\text{max}}=\text{min}(P_{req}(t),P_{m\text{max}})\\ u_{2\text{min}}=\text{max}(P_{req}(t)-P_{e\text{max}},-P_{m\text{max}}) \end{array}\right.$$
  where *u*_2max_ and *u*_2min_ represent the maximum power and minimum power allowed by the motor at time *t*, respectively.
9. Within the value range, the working power of the motor is discretized with a step size Δ*u*.
$$u_{2j}\in \left[u_{2\text{min}}(t):\Delta u:u_{2\text{max}}(t)\right],(j=1,2,\ldots ,k)$$ (29)
10. Calculate the Hamilton function value *H* (*t*, *u*_1*i*_, *u*_2*j*_) corresponding to each candidate control variable (*u*_1*i*_, *u*_2*j*_) according to Eq (22) until the *i* and *j* loops end.
11. Find the optimal control variables.
$${\left[u_{1},u_{2}\right]}^{*}=\text{arg}\;\text{min}\left(H\left(t,u_{1i},u_{2j}\right)\right)$$ (30)
12. According to the optimal control variables, the power battery status value is obtained. Determine whether the condition is met:
$$\left|SOC(t_{f})-SOC(t_{t\text{arg}et})\right|\leq \delta$$ (31)
  where *SOC* (*t*_*f*_) and *SOC* (*t*_*target*_) denote the final value and expected value of SOC, respectively; *δ* is a very small number.
  If Eq (31) is satisfied, the loop ends. Otherwise, reselect *ζ*_0_.
13. Adjustment of *ζ*_0_ value. In order to highlight the energy-saving effect, the final SOC values of the proposed strategy and the comparison strategy are set to be the same.
$$\Delta SOC=SOC(t_{f})-SOC(t_{t\text{arg}et})$$ (32)
  If Δ*SOC*<0, increase the value of *ζ*_0_. If Δ*SOC*>0, reduce the value of *ζ*_0_. This cycle continues until Eq (31) satisfies the conditions.

#### Control strategies used for comparison

Power following control strategy, as a rule-based, widely used, and mature control strategy, is usually used as a comparison strategy in energy-saving control. The PFCS used the rated power ratio of the motor and diesel engine as the power distribution ratio of the entire machine. The distribution proportion coefficient K is calculated as follows:

$$\text{K}=\frac{P_{erated}}{P_{erated}+P_{mrated}}$$ (33)

where K denotes the power distribution proportional coefficient; *P*_*erated*_ and *P*_*mrated*_ denote the rated power of the diesel engine and the motor, respectively.

In previous studies, the power following energy-saving control strategy has been described in detail. No more description here. The specific process can be found in literature [30].

## Results and discussion

Working condition prediction is of great significance for improving the energy efficiency and economy of tractors. Adaptive cubic exponential forecasting enables the smoothing coefficient to continuously follow changes in time series data and automatically adjust [31]. This method has better forecasting effects on time-varying data. The tractor velocity and plowing resistance change irregularly and show a nonlinear trend, so it is more suitable to use the adaptive cubic exponential forecasting method to predict. As an optimization algorithm that approximates global optimality, Pontryagin’s minimum principle has a fast calculation speed and can achieve better optimization results while ensuring calculation time. Therefore, this study uses Pontryagin’s minimum principle to optimize the prediction results in the prediction time domain to obtain better energy consumption economy.

**Fig 8:**
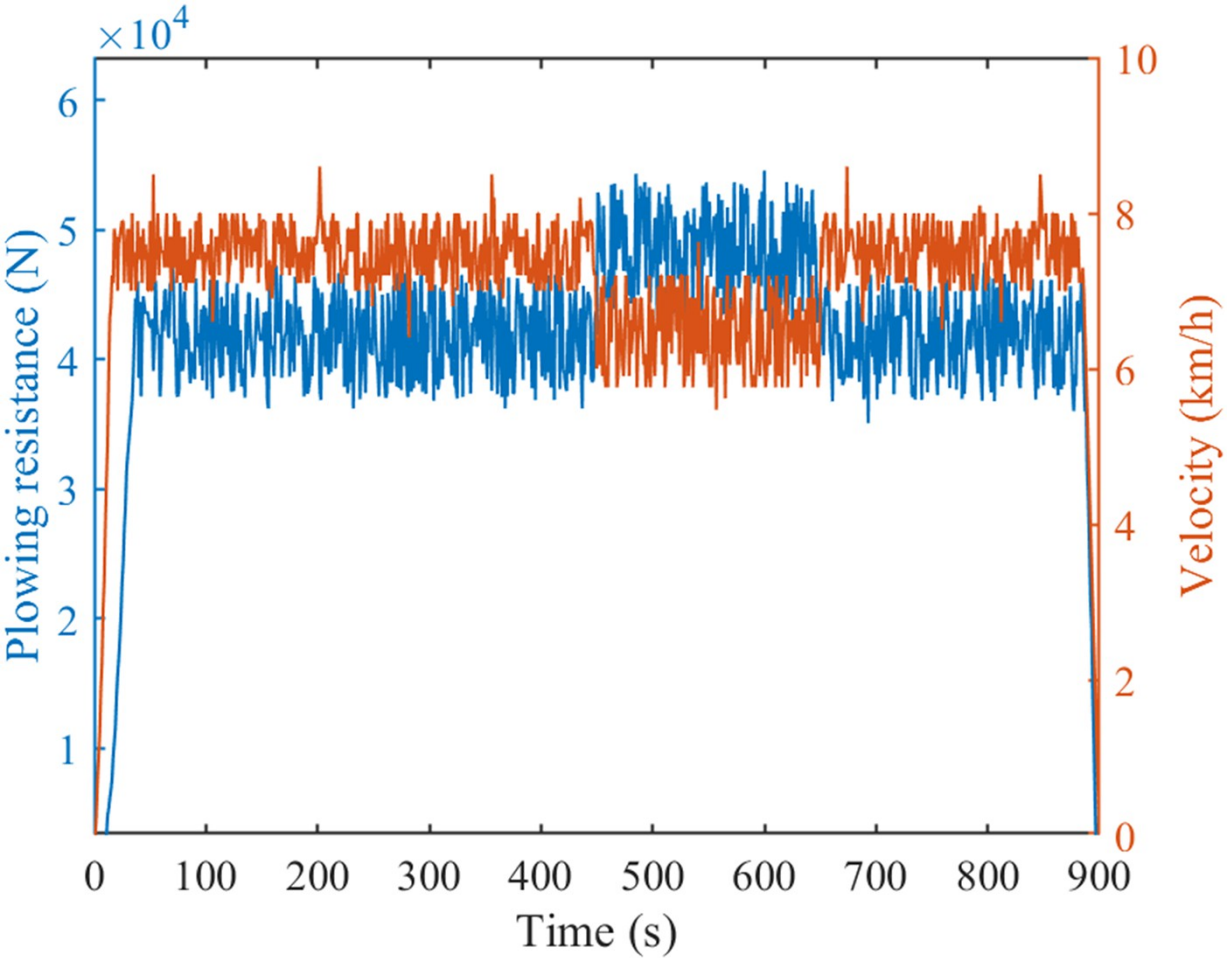
Tractor plowing working conditions diagram.

Plowing operation is one of the most representative working conditions of agricultural tractors. Based on the tractor field plowing operation test, the experimental data is input into the simulation model. Initialize all data before starting the simulation, and then input the tractor parameter information and measured operating condition information into the system, with a simulation step size set to one second. Especially, the variation of Lagrange multipliers *ζ* during the simulation process is sensitive to the total energy consumption cost of the tractor. In addition, during the simulation process, we neglect the influence of the external environment on the experiment. The plowing condition lasts for 900 seconds, and its operating velocity and farming resistance are shown in Fig 8. The operating velocity of the tractor is about 4.8 km/h, and the plowing resistance is about 42 kN. Especially, when the plowing resistance increases, the operating velocity will decrease.

**Fig 9:**
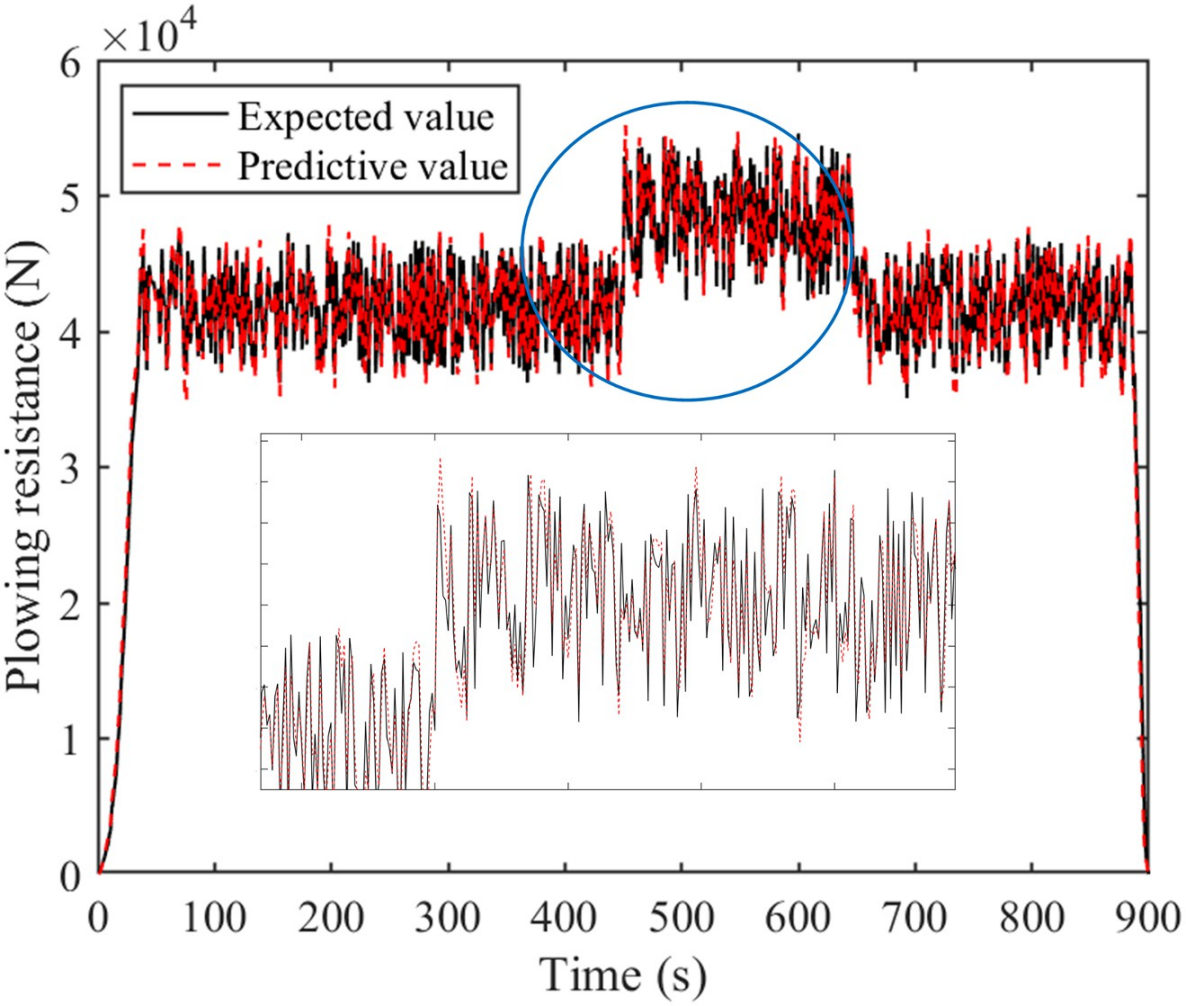
Comparison chart of plowing resistance prediction results.

**Fig 10:**
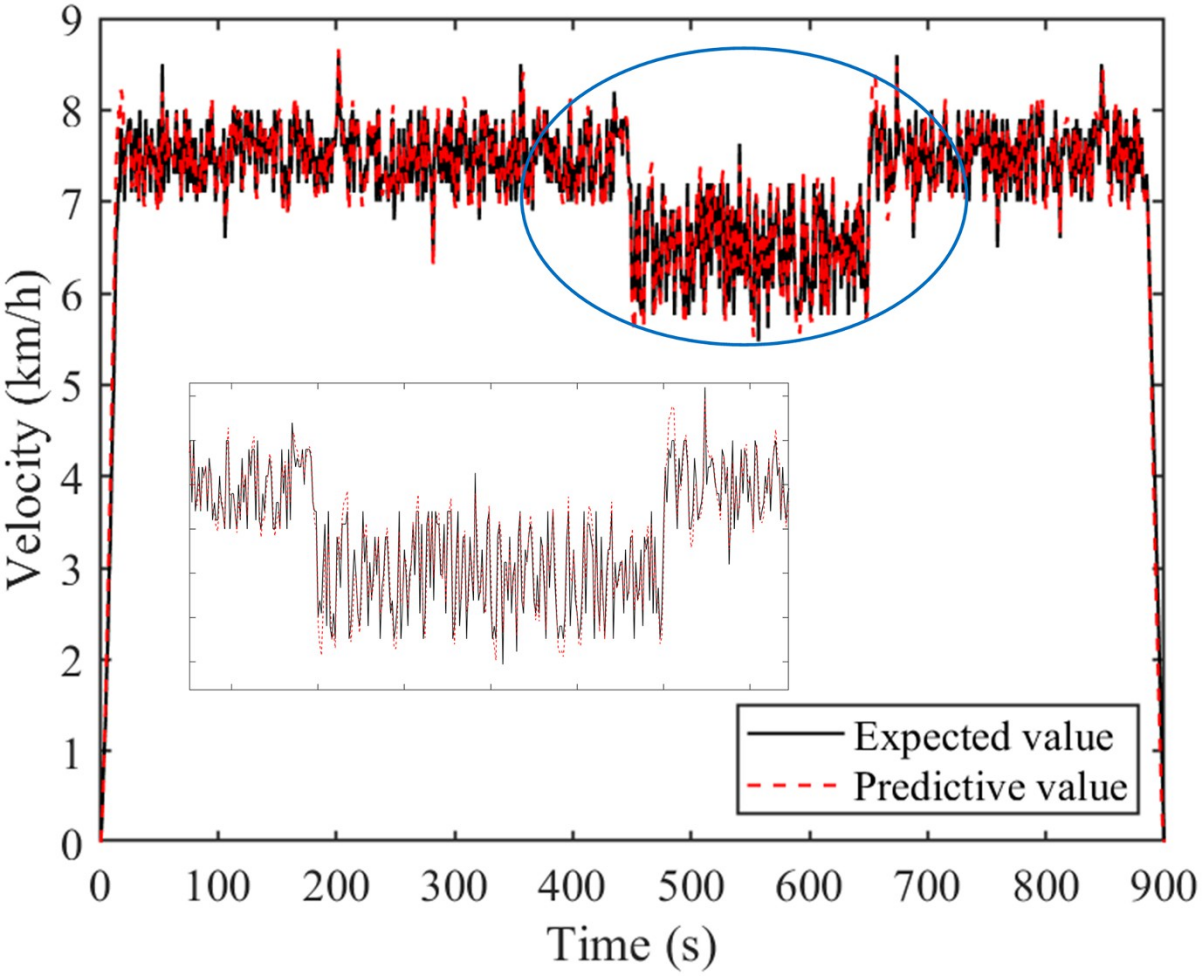
Comparison chart of plowing velocity prediction results.

**Fig 11:**
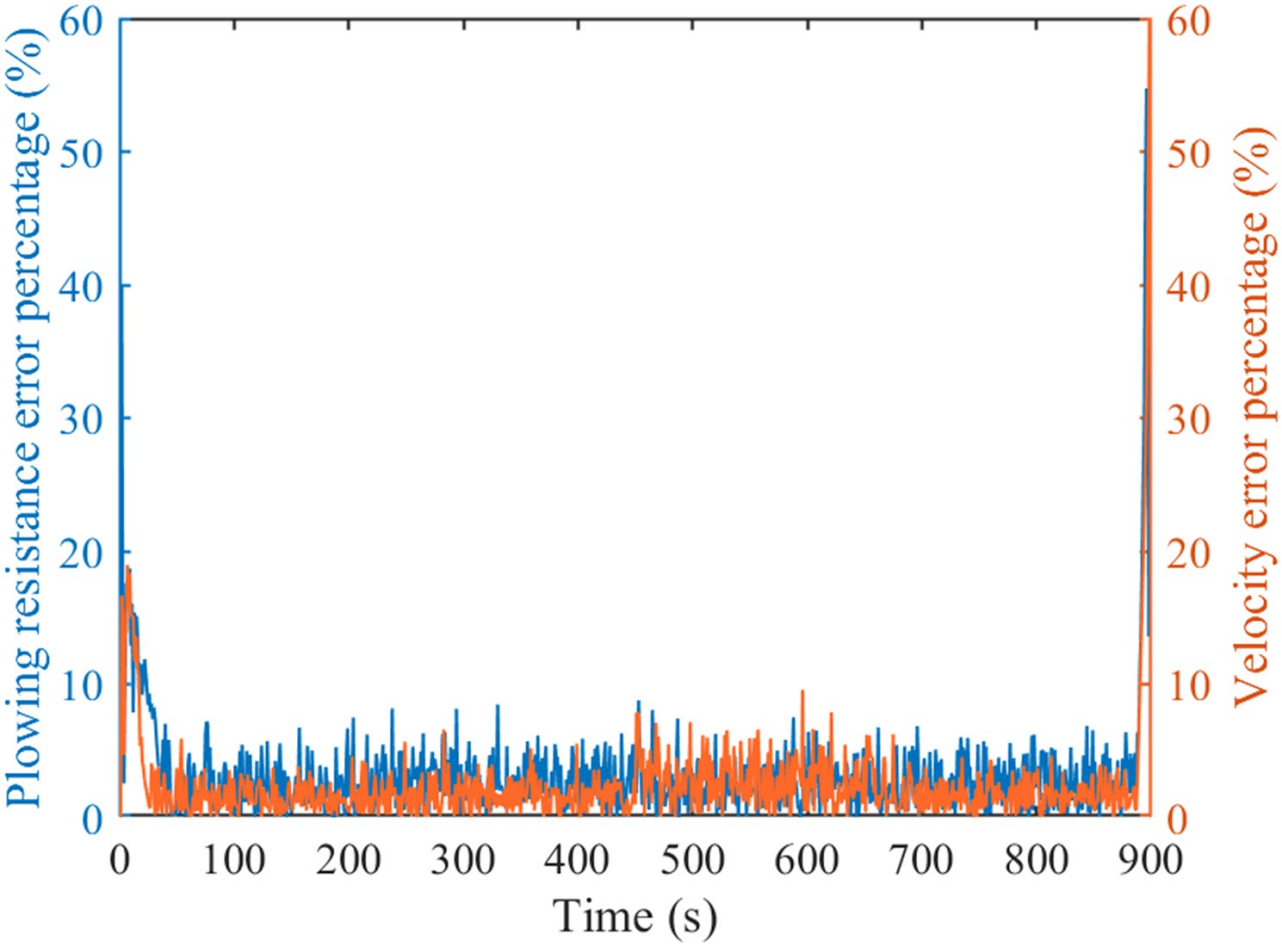
Forecast result error analysis chart.

Figs 9 and 10 are comparison charts of the prediction results of tractor resistance and velocity, respectively. As can be seen from the Figs, the prediction results are good and the expected value can be tracked. However, the prediction effect is poor when there is a sudden change in velocity or resistance. Specifically, at 450th and 620th seconds, there is a significant change in the predicted data, leading to an increase in prediction error. Fig 11 is a percentage plot of prediction error, represented by the absolute value of relative error. The relative error range is within 8%. The average relative error of velocity prediction and plowing resistance prediction are 2.48% and 3.20%, respectively. Especially, during the 450th and 620th seconds of tractor operation, there is a significant prediction error. This also corresponds precisely to the data mutation in Figs 9 and 10, respectively. However, the error is larger during the starting and stopping phases of the tractor, reaching 50%. This is due to the poor prediction effect of the adaptive cubic exponential forecasting method on linear mutation data. Overall, the error is within the allowable range and meets the accuracy requirements.

**Fig 12:**
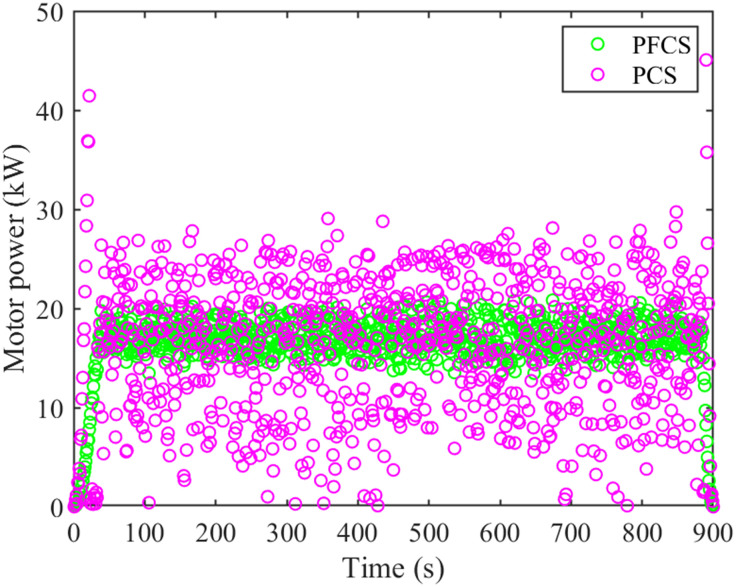
Motor power comparison chart.

**Fig 13:**
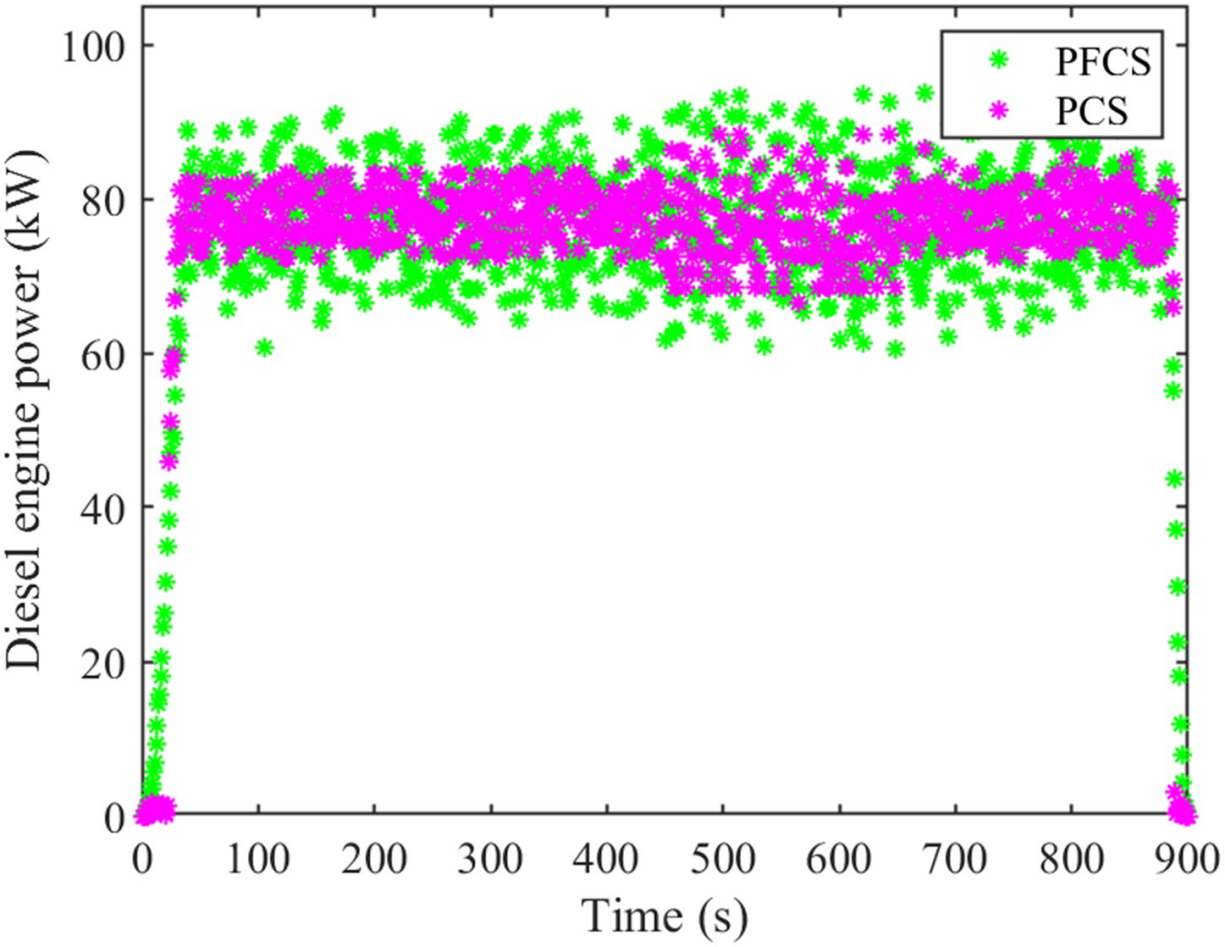
Diesel engine power comparison chart.

**Fig 14:**
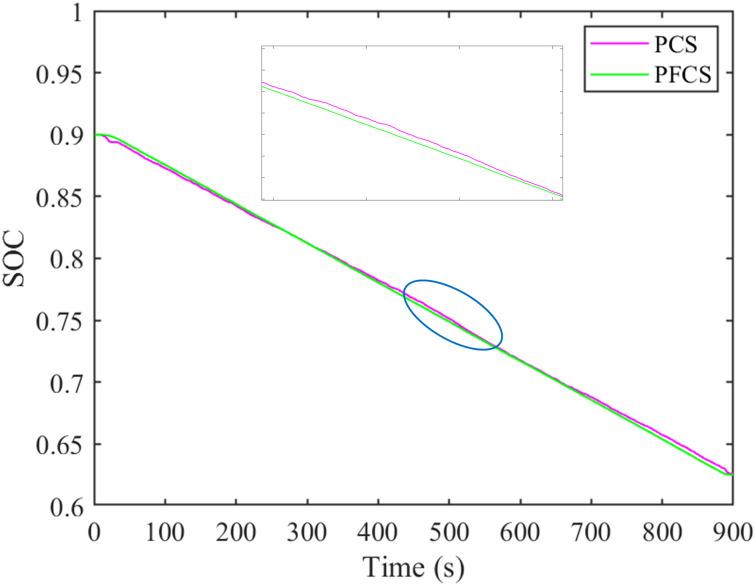
SOC change comparison chart.

**Fig 15:**
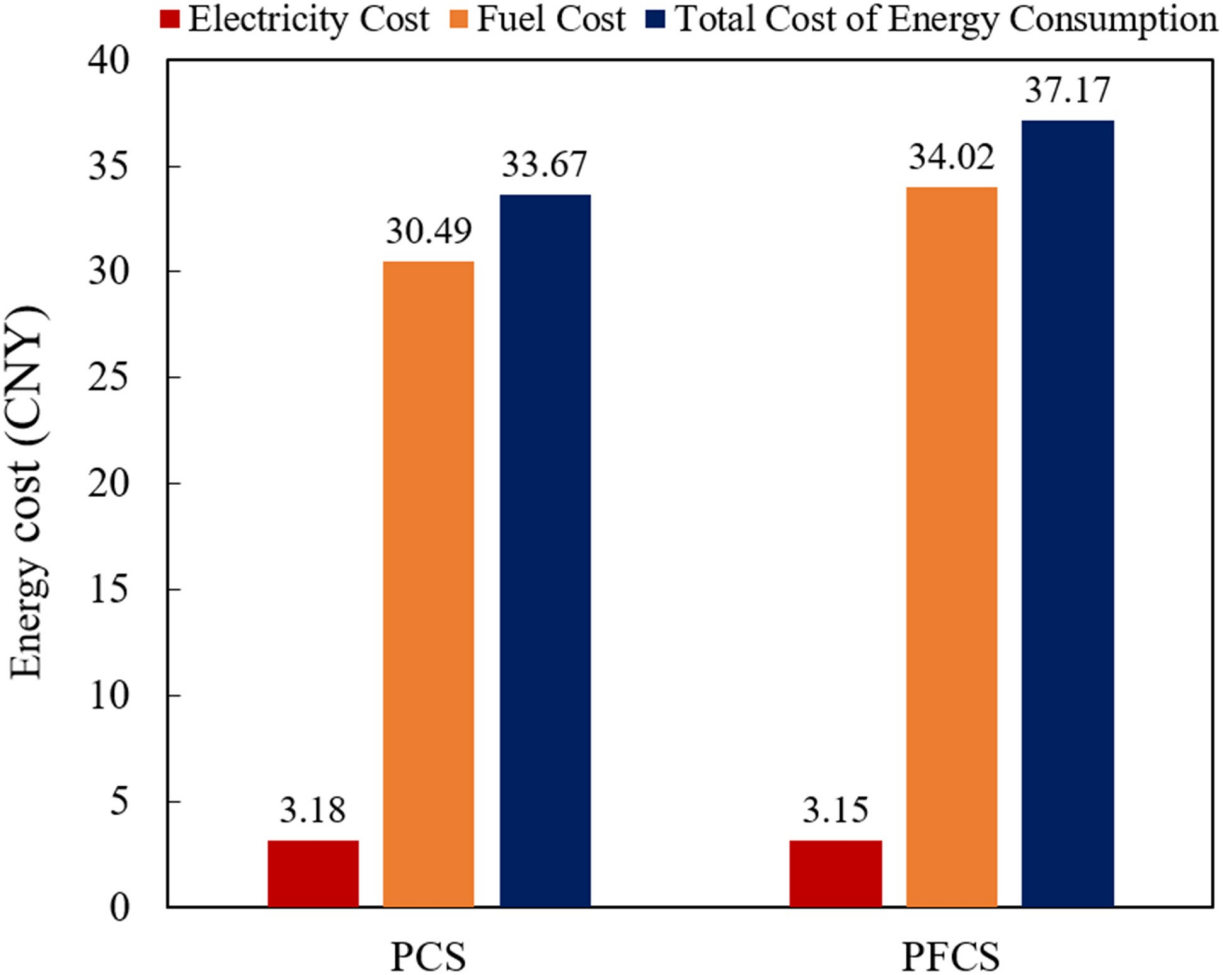
Tractor energy consumption cost analysis chart.

During tractor plowing operation, under the PCS and PFCS, the motor and diesel engine power are shown in Figs 12 and 13. The change of battery SOC status value is shown in Fig 14. The total cost of energy consumption is shown in Fig 15.

It can be seen from Figs 12 and 13 that under the two control strategies, the motor does not have negative power. Under the PCS, the motor power distribution is dispersed. At the start and end of tractor operation, part of the working power exceeds the rated power of the motor. The power of diesel engine is relatively centralized, about 70–80 kW. Under the PFCS, the motor power is relatively concentrated, around 15–20 kW. The working power range of diesel engines is relatively large, about 65–95 kW. From Fig 14, it can be seen that under the PCS, the SOC of the power battery shows a downward trend and is relatively gentle. There is no increase, but it shows a fluctuating curve of decline. The final SOC value is 0.626. Under the PFCS, the SOC of the power battery decreases linearly without any fluctuations. The final SOC value is 0.625. The changes in SOC curves under the two control strategies also correspond precisely to the changes in motor power. The larger amplitude of motor power variation, the more frequent it becomes, and the SOC curve also fluctuates and changes accordingly. On the contrary, if the motor power changes within a small range, the SOC curve will approximately decrease in a straight line. At last, from Fig 15, it can be seen that the electricity cost under the two control strategies is almost the same. However, fuel costs vary greatly. The total energy costs of the PFCS and the PCS are 37.17 China Yuan (CNY) and 33.67 CNY, respectively. Under the proposed control strategy, the total energy costs diminished by 9.42%.

**Fig 16:**
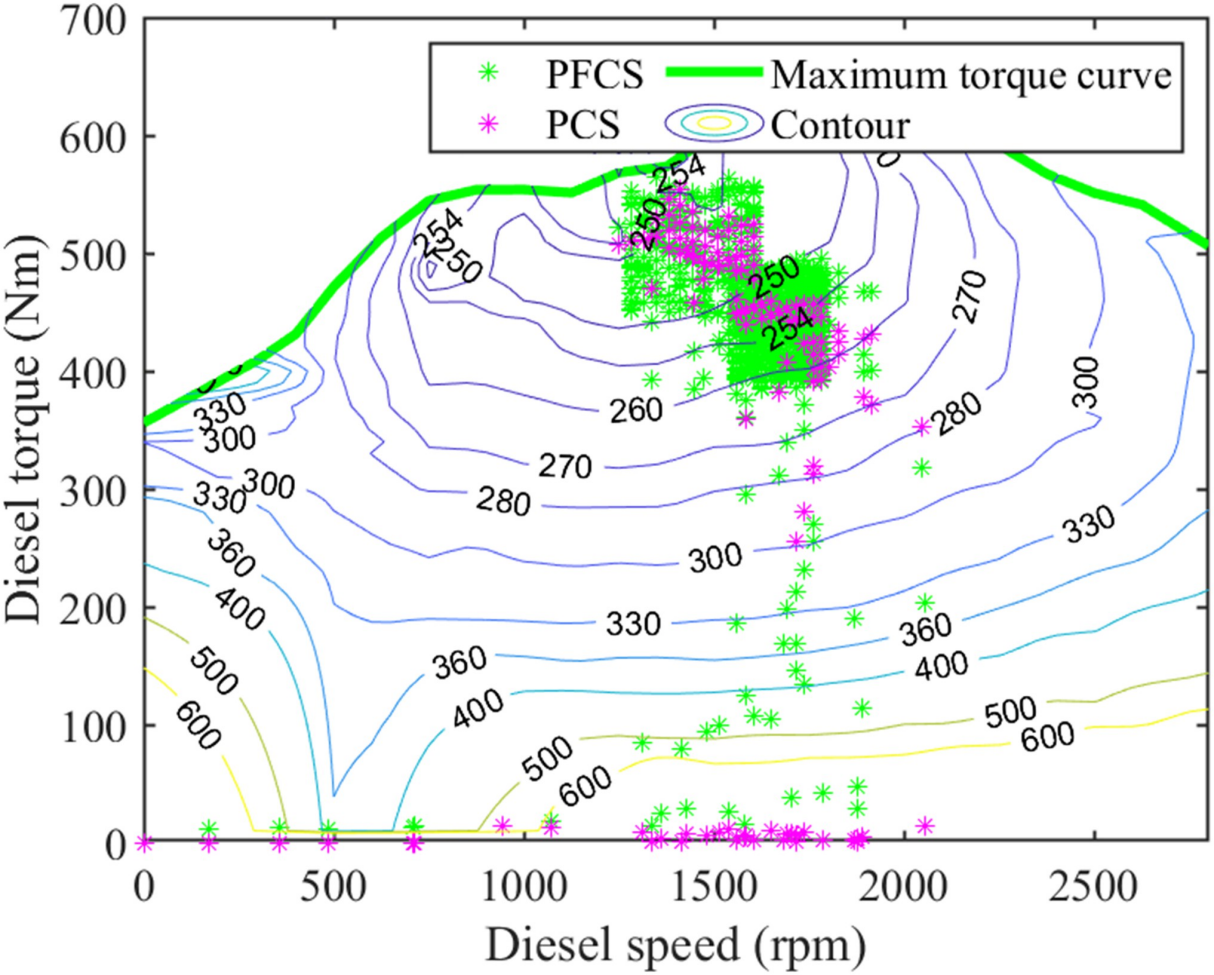
Diesel engine map for plowing operations.

**Fig 17:**
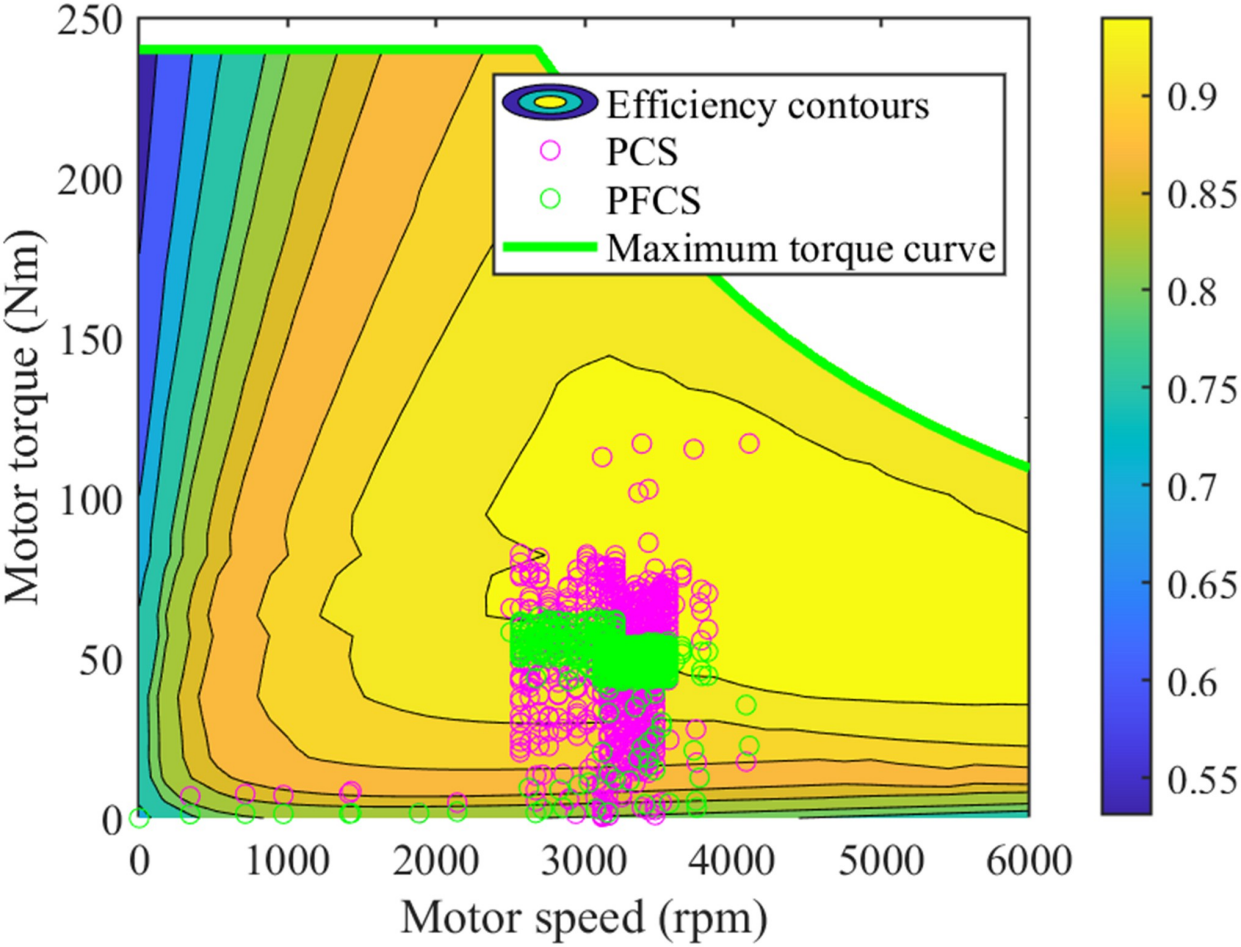
Motor map for plowing operations.

The Diesel engine map and motor map under the two control strategies for plowing operations are shown in Figs 16 and 17.

It can be seen from Figs 16 and 17 that under the PCS, the working points of the diesel engine are relatively centralized. Moreover, the work points are all located in high-efficiency areas. The working points of the motor are relatively scattered. However, most of its work locations are also in high-efficiency areas. The overall work efficiency is higher. Under the PFCS, the diesel engine has a larger working range. And the efficiency of some work points is very low. However, the working points of the motor are centralized, and it has high work efficiency. However, under the PFCS, the working efficiency of the diesel engine is ignored, and it is impossible to optimize and adjust the working status of the motor and diesel engine according to the operating conditions, resulting in lower overall working efficiency.

In summary, through the analysis of simulation results of plowing operation conditions, it can be concluded that: The PCS can reasonably allocate the working torque of the motor and diesel engine, so that the motor and diesel engine can run in a high-efficiency zone. The tractor has high working efficiency. However, the PFCS can only allocate motor and diesel engine power according to fixed ratio. It is impossible to make corresponding power allocation according to the tractor operating conditions. The working efficiency of the tractor is low. Therefore, compared with the PFCS, the proposed control strategy effectively increases the energy utilization and increases the energy consumption economy of the tractor.

## Conclusions

In order to improve the energy efficiency of tractors, this paper proposes an energy-saving control strategy based on Pontryagin’s minimum principle that integrates working condition prediction for agricultural hybrid tractors. Firstly, determine the parameters of each component of the drive system based on the transmission scheme of the agricultural hybrid tractor. Secondly, model its main components and build a simulation model of the tractor. Furthermore, aiming at the energy-saving control problem of hybrid tractors, a predictive control strategy based on Pontryagin’s minimum principle integrating working condition prediction is proposed. The PCS is an integration of adaptive cubic exponential forecasting method and Pontryagin’s minimum principle. Finally, in order to verify the superiority of the proposed control strategy, PFCS was used for comparison and validated based on the MATLAB simulation platform. The research results are as follows:

1. The use of adaptive cubic exponential forecasting method has shown good prediction results for tractor plowing conditions. The relative error range is within 8%. The average relative error of velocity prediction and plowing resistance prediction are 2.48% and 3.20%, respectively.
2. Under plowing conditions, the total energy costs of the PFCS and the PCS are 37.17 CNY and 33.67 CNY, respectively. The total energy costs diminished by 9.42%.

Currently, we have only studied tractor plowing conditions. After, we will study the rotary tillage and transportation conditions of tractors. Subsequently, we will design a new control strategy framework that may include neural networks to meet various operating conditions. In addition, the adaptive cubic exponential forecasting method is poor at predicting linear mutation inflection points. The reader can further increase the preciseness of the working condition prediction by changing the prediction method or adopting intelligent prediction algorithms.

## Supporting information

S1 File Information on tractor plowing conditions and prediction results data. (ZIP)
